# Phylogeography of the Grassland Skipper *Ochlodes venatus* Supports the Hypothesis That This Japanese Grassland Insect Has a Long History

**DOI:** 10.1002/ece3.74193

**Published:** 2026-08-18

**Authors:** Naoyuki Nakahama, Gohta Kinoshita, Masato Hayamizu, Kazusa Nishimura, Seiya Kudo, Yuriy Tshistjakov, Atsushi Ohwaki

**Affiliations:** ^1^ Graduate School of Agriculture Osaka Metropolitan University Sakai Osaka Japan; ^2^ Faculty of Science, Academic Assembly University of Toyama Toyama Japan; ^3^ Forestry Research Institute Hokkaido Research Organization Bibai Hokkaido Japan; ^4^ Graduate School of Environmental, Life, Natural Science and Technology Okayama University Okayama‐shi Okayama Japan; ^5^ Faculty of Agriculture and Life Sciences Hirosaki University Hirosaki Aomori Japan; ^6^ Federal Scientific Center of the East Asia Terrestrial Biodiversity Far East Branch Russian Academy of Sciences Vladivostok Russia; ^7^ College of Arts and Sciences J. F. Oberlin University Machida Tokyo Japan

**Keywords:** East Asia, genome‐wide SNPs, MIG‐seq, mitochondrial DNA, skipper

## Abstract

In Japan, with its warm and humid climate, the conservation value of grassland ecosystems has often been underestimated, as they were believed to mainly have been maintained by human activities. It is thus essential to understand when grassland organisms first colonized Japan and have maintained since then to understand the history of grassland ecosystems in Japan. Here, we conducted a phylogeographical study using genome‐wide single nucleotide polymorphisms from MIG‐seq and mitochondrial DNA of *Ochlodes venatus* (Lepidoptera: Hesperiidae), a grassland skipper species, in Japan and eastern Russia. Genetic analyses showed that the *O. venatus* groups in northern and western Japan diverged 1.5 million years ago and then hybridized approximately 100,000 years ago. It was also found that *O. venatus* populations have been maintained in Japan for at least 200,000 years. The long‐term demographic history inferred within Japan is notably longer than that of other grassland insects as estimated in previous studies. The results demonstrated that this grassland butterfly has been present long before human colonization in Japan, and that the Japanese grasslands have been naturally maintained over a long period, highlighting the conservation value of *O. venatus* in Japan.

## Graphical abstract

The key finding of this manuscript is that *Ochlodes venatus*, a species of Japanese grassland butterfly, inhabited Japan far earlier than previously known. This study estimates *O. venatus* was distributed in Japan at least 200,000 years ago. This provides crucial evidence that grassland organisms inhabited Japan much earlier than previously thought.

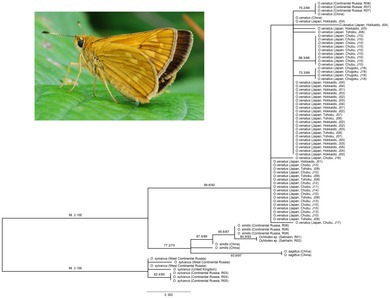

## 1 Introduction

Seminatural grassland ecosystems, which are maintained through anthropogenic management practices such as mowing, grazing, and burning, have high biodiversity (Ushimaru et al. 2018). Studies have estimated that humans first colonized Japan approximately 32,000–38,000 years ago (Gakuhari et al. 2020; Jinam et al. 2015), and it has been asserted that human disturbances increased the area of temperate grasslands after the Jomon period (~16,000 years ago; Ogura 2006). It is estimated that, at the beginning of the 20th century, approximately 17% of the country was covered by seminatural grassland, which was considered a relatively common ecosystem (Ogura 2006). However, given that Japan has a warm and humid temperate climate, the climax vegetation in many areas was considered to be forest (Miyawaki et al. 1975). Therefore, grassland ecosystems have been relatively overlooked from a conservation perspective, despite many endangered species inhabiting seminatural grasslands in Japan (Ohwaki 2018; Yamaki and Shibasaki 2023). To appreciate grasslands as an inherent part of the Japanese ecosystem, it is necessary to understand whether they could have existed in the Japanese archipelago during the warm interglacial periods prior to the arrival of humans.

The critical status of seminatural grasslands in Japan is also a substantial motivation for performing phylogeographic studies of the country's grassland organisms. Over the last 100 years, the grassland area has decreased to less than one‐tenth of its original size, and grassland biodiversity has also deteriorated significantly due to conversion to artificial pastures, damage by sika deer ( *Cervus nippon* ), and the abandonment of management practices (Ogura 2006; Ushimaru et al. 2018). As a result, many seminatural grassland organisms are listed as endangered species in Japan (Nakahama et al. 2023). Once genetic diversity of endangered organisms is lost, it is difficult to estimate their history due to the associated loss of genetic information (Nakahama and Isagi 2018). The loss of such information also has significant disadvantages for conservation genetics studies. For example, *Melitaea protomedia*, one of the most critically endangered butterfly species in Japan, has been shown to have undergone significant changes in its genetic structure due to a decline in genetic diversity (Nakahama and Isagi 2018). Thus, to construct conservation strategies, genetic analyses need to be performed before populations and genetic diversity experience serious declines.

**FIGURE 1:**
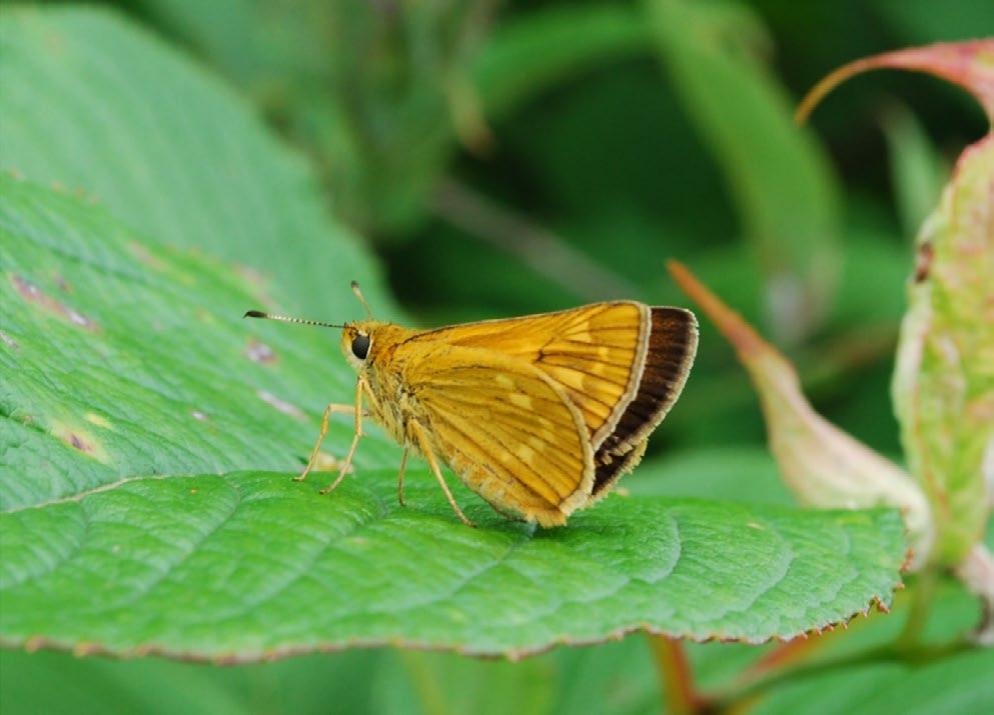
The photograph of *Ochlodes venatus* in Japan.

*Ochlodes venatus* (Hesperiidae, Figure 1) is distributed in southeastern Russia, northeastern China, the Korean Peninsula, and Japan (Shirôzu 2006). In Japan, this species is mainly distributed in seminatural grasslands on Hokkaido and the Honshu Islands, and its larvae primarily feed on Poaceae grasses (Shirôzu 2006). It is listed as “endangered” on the Red List of seven prefectures and as “near threatened” on the Red List of two prefectures in Japan, although it is not listed in the Japanese Red List (search system of Japanese Red Data, http://jpnrdb.com/index.html). Meanwhile, three closely related species (*venatus*‐complex) from continental East Asia have been reported, namely, *O. sagittus* , *O. similis* , and *O. sylvanus*, none of which are found in Japan. Since the taxonomic classification and distribution of these species on the Eurasian continent are still under debate (Chiba and Tsukiyama 1996; Farahpour‐Haghani et al. 2023; Paek and Shin 2014; Zhu et al. 2023), it is anticipated that genetic analyses would lead to novel taxonomic and distributional findings if specimens belonging to multiple *venatus*‐complex species from East Asia are evaluated. Although other *Ochlodes* species, *O. asahinai*, and *O. ochraceus* (the Japanese population was restored as *O. rikuchiana* in Zhu et al. (2023)) also distribute in Japan, they belong to separate species complexes, leaving *O. venatus* as the unique representative of its specific complex in the Japanese archipelago (Chiba and Tsukiyama 1996).

In this study, we clarified the phylogeographic history of *O. venatus* in Japan and the Russian Far East in order to understand the origin and history of grassland species in Japan through genome‐wide single nucleotide polymorphism (SNP) analysis. We also provide valuable information for the future conservation of this species, the population of which is at substantial risk of decline. In addition, our genetic analysis of the species and its relatives across Japan, Sakhalin, and the Russian Far East provides novel insights into their distributions and introgression of this species complex.

## 2 Materials and Methods

### 2.1 Sampling and DNA Extraction

**FIGURE 2:**
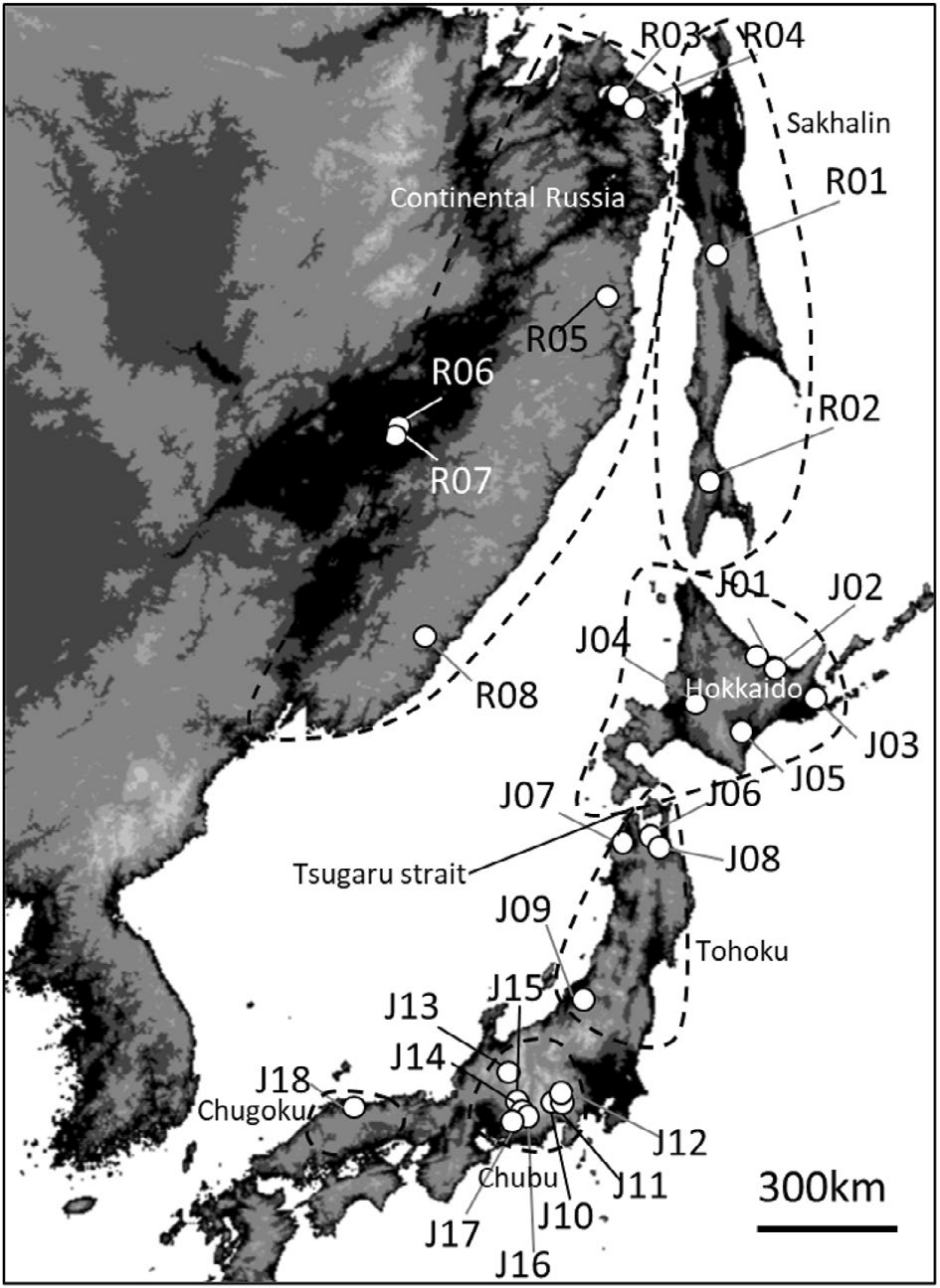
Map of East Asia and location of the sampling sites of *Ochlodes* species belonging to the *venatus*‐complex.

A total of 57 *O. venatus* individuals were collected at 18 sites in Japan (Figure 2 and Table 1). In continental Russia and Sakhalin, we also collected 11 individuals at 8 sites (Figure 2 and Table 1). We attempted to collect samples of *O. venatus* in South Korea, but as this species is classified as vulnerable (VU) in South Korea (National Institute of Biological Resources 2014), we decided not to proceed with the collection. We were also unable to collect samples of *Ochlodes* spp. from China.

**TABLE 1:** Species name and genetic diversity measurements of each *Ochlodes* species in Japan and Russia.

Site ID	Species (based on mtDNA)	Species (based on MIG‐seq)	Country	District	Latitude	Longitude	*N*		*H*_O_	*H*_S_
							mtDNA	MIG‐seq		
J01	*Ochlodes venatus*	*Ochlodes venatus*	Japan	Hokkaido	44.052	143.504	3	3	0.042	0.061
J02					43.872	143.914	4	4	0.047	0.069
J03					43.318	145.146	7	7	0.049	0.068
J04					43.257	141.840	6	6	0.044	0.063
J05					42.627	143.291	1	1	0.046	*—*
J06				Tohoku	40.697	140.927	1	1	0.044	*—*
J07					40.624	140.278	2	2	0.053	0.081
J08					40.290	141.079	1	1	0.045	*—*
J09					37.380	139.267	6	6	0.052	0.085
J10				Chubu	35.448	138.613	13	13	0.052	0.083
J11					35.421	138.821	1	1	0.043	*—*
J12					35.581	138.754	1	1	0.058	*—*
J13					36.225	137.537	3	3	0.055	0.085
J14					35.351	137.677	1	1	0.053	*—*
J15					35.247	137.713	1	1	0.046	*—*
J16					35.142	137.920	1	1	0.046	*—*
J17					35.284	137.570	1	1	0.052	*—*
J18				Chugoku	35.370	133.517	4	4	0.040	0.060
R01	*Ochlodes similis*	*Ochlodes venatus*	Russia	Sakhalin	50.955	142.662	1	1	0.036	*—*
R02	*Ochlodes similis*	*Ochlodes venatus*			47.066	142.519	1	1	0.046	*—*
R03	*Ochlodes sylvanus*	*Ochlodes sylvanus*		Continental Russia	53.411	140.045	1	1	0.044	*—*
R04	*Ochlodes sylvanus*	*—*			53.105	140.320	1	*—*	*—*	*—*
R05	*Ochlodes sylvanus*	*Ochlodes sylvanus*			50.530	139.524	1	1	0.063	*—*
R06	*Ochlodes similis*	*—*			48.181	135.093	1	*—*	*—*	*—*
R07	*Ochlodes venatus*	*Ochlodes venatus*			48.153	135.032	2	2	0.047	0.138
R08	*Ochlodes venatus*	*Ochlodes venatus*			44.383	135.283	1	1	0.049	*—*
	*Ochlodes similis*	*Ochlodes similis*			44.383	135.283	2	2	0.033	0.077

Abbreviations: —, no data; *H*_O_, observed heterozygosity; *H*_S_, expected heterozygosity; *N*, number of samples.

Because these samples potentially included *O. venatus*, *O. similis* , and *O. sylvanus*, which were difficult to differentiate morphologically during the field survey, we identified them based on mitochondrial DNA sequences and MIG‐seq analyses. Genomic DNA was extracted from the middle or hind leg using the Chelex method (Miura et al. 2017).

### 2.2 Mitochondrial DNA Sequencing Analysis

To sequence mitochondrial DNA, we used 68 samples from Japan and Russia. One primer pair was used to amplify 667 base pairs of the COI gene: LepF (5′‐ATTCAACCAATCATAAAGATATTGG‐3′) and LepR (5′‐TAAACTTCTGGATGTCCAAAAAATCA‐3′) (Hebert et al. 2004). PCR was performed in a final reaction volume of 10 μL, containing 1 ng of template DNA, 5 μL of KOD One PCR Master Mix (Toyobo, Osaka, Japan), and 0.15 μL of each primer at 20 μM. The PCR cycling conditions were as follows: template denaturation at 95°C for 5 min; 35 cycles of denaturation at 94°C for 10 s, annealing at 50°C for 5 s, and extension at 72°C for 15 s; and final extension at 72°C for 5 min. After checking the presence and quality of amplicons by electrophoresis on 1% agarose gels, bidirectional sequencing was performed at Eurofins Genomics (Tokyo, Japan) using the same primers as for PCR. The mitochondrial sequencing data were aligned using ClustalW (Thompson et al. 1994) and an algorithm implemented in MEGA X (Kumar et al. 2018). Our sequences were registered in GenBank (Table 2). The haplotypes at each site were identified using MEGA X (Kumar et al. 2018).

**TABLE 2:** Prior distributions in DIYABC‐RF analysis for four populations of *O. venatus* in Japan.

Parameter	Minimum	Maximum
Effective population size		
N1 (Hokkaido)	10	2,000,000
N2 (Tohoku)	10	2,000,000
N3 (Chubu)	10	2,000,000
N4 (Chugoku)	10	2,000,000
Na	10	5,000,000
Nb	10	5,000,000
Nc	10	5,000,000
Time scale in generations		
t1	10	1,000,000
t2	10	1,000,000
t3	10	2,000,000
Admixture		
ra	0.001	0.999

We also added the COI sequences of two *O. venatus* in China (GenBank accession numbers: OQ452925 and OQ4529262; Zhu et al. 2023), two *O. similis* in China (GenBank accession numbers: OQ452929 and OQ452930; Zhu et al. 2023), two *O. sagittus* in China (GenBank accession numbers: OQ452931 and OQ452932; Zhu et al. 2023), and three *O. sylvanus* in Russia (GenBank accession numbers: PQ611781–PQ611783) as an outgroup from GenBank. Since Zhu et al. (2023) identified all species based on detailed morphological data, we concluded that these identification results are reliable.

The maximum likelihood (ML) tree was calculated using IQ‐Tree version 2.2.0 (Minh et al. 2020) with an ultrafast bootstrapping (UB) and SH‐aLRT branch test conducted with 1000 replicates. Estimation of substitution models was performed using ModelFinder in IQ‐Tree version 2.2.0 (Minh et al. 2020) based on the Bayesian information criterion (BIC) value. The selected COI model was HKY + F + I. The estimated tree was visualized and edited with FigTree version 1.4.3.

### 2.3 MIG‐Seq Analysis

We conducted a MIG‐seq (multiplexed ISSR genotyping by sequencing) analysis for all species across Russia and Japan, to elucidate the high‐resolution genetic diversity and phylogenetic relationships based on the nuclear genome (Suyama and Matsuki 2015; Suyama et al. 2022). First, we amplified the ISSRs from genomic DNA using eight pairs of multiplex primers, designated “MIG‐seq primer Set 1,” according to the protocol for KOD One PCR Master Mix (Toyobo Co. Ltd.) by Nishimura et al. (2024). We diluted the first PCR product 20 times and used it as the template DNA for the second PCR to build an indexed library for the Illumina sequencing platform, using the “second PCR primers” as described by Nishimura et al. (2024). Then, each PCR product was pooled into a single library, purified with AMPure XP beads (Beckman Coulter Life Sciences, Tokyo, Japan), and reconditioning PCR was performed according to the protocol of Nishimura et al. (2024). The reconditioning PCR was modified as follows: 98°C for 60 s, 60°C for 30 s, 72°C for 30 s, and final extension at 72°C for 1 min. The size of the reconditioning PCR products was selected (300–600 bp) using SPRIselect (Beckman Coulter, Tokyo, Japan). The library was sequenced on a DNBSEQ‐G400 (MGI Tech Co. Ltd., Tokyo, Japan) with 150 bp paired‐end reads by Fasmac Co. Ltd. (Atsugi, Japan).

To trim Illumina adapter sequences and low‐quality reads, we used Trimmomatic ver.0.39 (Bolger et al. 2014). Trimmed reads were processed using the following parameters, keeping paired‐end reads: SLIDINGWINDOW:4:15, CROP:150, HEADCROP:17, LEADING:20, TRAILING:20, and MINLEN:79. Processed reads for each sample were mapped to the reference genome (species name: *Ochlodes sylvanus* and genome accession: GCA_905404295.2) assembled by Lohse et al. (2023) using the Burrows–Wheeler Aligner (BWA) program v.2.2.1 (Li and Durbin 2009) and the BWA‐MEM algorithm with the default parameters. The SAM files obtained by read mapping were converted into BAM files and sorted using SAMtools (Li et al. 2009). For population genetic analysis, we used “ref_map.pl” and “populations” in Stacks v2.68 software (Catchen et al. 2013; Rochette et al. 2019), with the following option settings: minimum proportion of individuals required to process a locus across all data (*R*) = 0.8, minimum minor allele count required to process an SNP (min_mac) = 2, maximum observed heterozygosity needed to process a nucleotide site at a locus (max_obs_het) = 0.6, and data analysis restricted to a single SNP per locus (write_single_snp). For sample selection, TASSEL ver. 5.2.70 (Bradbury et al. 2007) was used with the following setting: minimum proportion of sites present = 0.5.

### 2.4 Estimating Genetic Structure and Diversity Based on MIG‐Seq

We performed principal component analysis (PCA) using TASSEL ver. 5.2.70 (Bradbury et al. 2007). As the clustering analysis model assumed linkage equilibrium among markers (Alexander et al. 2009), we used only one SNP per locus, as described earlier. We also estimated the molecular phylogenetic network tree using the NeighborNet algorithm (Bryant and Moulton 2004) in the SplitsTree App. v. 6.4.11 (Huson and Bryant 2024) based on the concatenated data of all SNP sequences.

We conducted clustering analysis using the software Admixture (Alexander et al. 2009), assuming between 1 and 12 ancestral populations (*K*), and performed 10 replicate runs per *K* value. To confirm the cluster assignment in *O. venatus*, we used only samples from Japan and Sakhalin. This is because, according to the results of NeighbourNet and PCA based on the MIG‐seq, the individuals collected from the continental Russia were genetically distinct from those in Japan and Sakhalin (see Section 3). The individuals assigned to each cluster with an assignment probability of > 0.90, using the criteria of Nakahama et al. (2019), were identified as each *Ochlodes* species. We calculated codominant genotypic distance using the covariance standardized method (Smouse and Peakall 1999) among the individuals from Japan and Sakhalin from the SNP dataset, using GenAlEx v.6.5.03 (Peakall and Smouse 2006) as the genetic distance index. A Mantel test (Mantel 1967) was used to assess correlations between genetic distance (codominant genotypic distance) and geographical distance among all collection sites, with 9999 permutations performed in GenAlEx v.6.5.03 (Peakall and Smouse 2006). The individuals collected in continental Russia were excluded from the Mantel test.

We evaluated the genetic diversity in terms of the observed heterozygosity (*H*_O_) and expected heterozygosity (*H*_S_) for each population using the hierfstat package in R ver. 4.0.4 (R Development Core Team 2021). Among the indicators of genetic diversity, *H*_S_ is corrected for sample size differences.

### 2.5 Demographic Analysis Based on MIG‐Seq

To assess possible scenarios explaining the genetic divergence among *O. venatus* populations on Hokkaido and Honshu Islands, we performed approximate Bayesian computation (ABC) using a random forest approach (Breiman 2001) with DIYABC‐RF v.1.2.1 (Collin et al. 2021). Based on the results of the Admixture and NeighborNet analyses mentioned above, we defined the Hokkaido samples as a single independent population. We divided the Honshu samples into three populations: the Tohoku, Chubu, and Chugoku populations. We filtered SNP loci using TASSEL to ensure that at least half of the individuals from each population were included. The remaining 3334 SNPs from the four populations were used for the DIYABC‐RF analysis. We tested 11 scenarios by assuming simple divergence among the four populations without admixture (Scenarios 1–5) or with a single admixture event for a specific population (Scenarios 6–11) (Figure S1). We used prior settings of the parameters for effective population sizes and the numbers of generations listed in Table 2. All previous values were drawn from uniform distributions, and an additional condition was applied to specify t1 < t2 and t2 < t3. We assumed changes in the effective population size of the ancestral population before the oldest split at t3 (i.e., Na) in all scenarios, as well as in the past populations at t1 or t2 (i.e., Nb and Nc), to improve the efficiency of the simulation. Using all summary statistics obtained from 20,000 simulations per scenario, as well as the optional axes of linear discriminant analysis, a random forest estimate was conducted to determine the best‐fit model. The number of trees in the random forest was set to 1000. The best‐fit model was chosen based on the number of classification votes, and then the posterior probability for that scenario, as well as global and local error rates, was estimated to assess the chosen scenario and the quality of the prediction. Then, we re‐ran the DIYABC‐RF analysis exclusively for the best model to estimate its parameters under the same prior setting in Table 2. The summary statistics were derived from 100,000 simulations under this model. We assumed a generation time of 1 year for *O. venatus* as adults only emerge once a year.

## 3 Results

### 3.1 Phylogeny Based on Mitochondrial DNA Sequences

**FIGURE 3:**
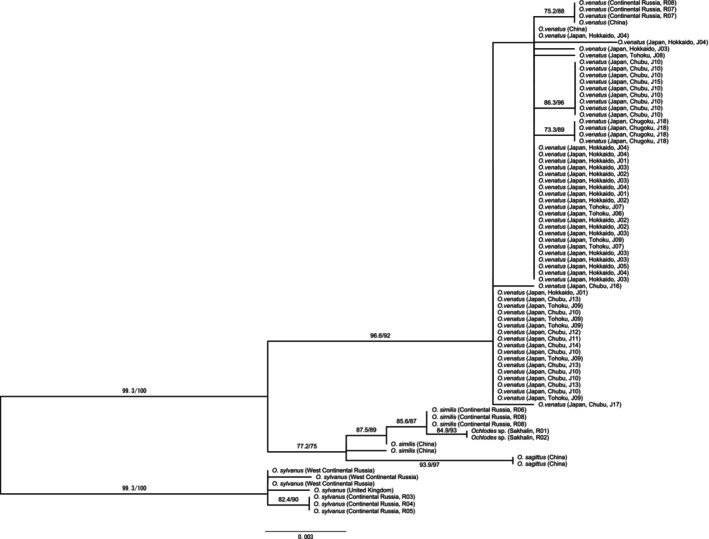
The phylogenetic tree of the specimens investigated in this study. The maximum likelihood (ML) tree for mitochondrial COI markers of 667 bp. The node numbers represent SH‐aLRTs and ML UB support values. SH‐aLRT < 70 and UB < 70 are denoted by “‐”.

We determined the COI sequences (667 bp) from 68 samples collected at 26 sites and obtained COI sequences of 9 samples from GenBank. The phylogenetic relationships of 77 *Ochlodes* individuals were estimated, with a mean likelihood value of −1108.100035 (Figure 3). *Ochlodes venatus* belongs to a supported monophyletic clade (UB = 92%, SH‐aLRT = 96.6%) that contains individuals collected in Japan, China, and the eastern continental regions of Russia. The clade of *O. sylvanus* was also supported (UB = 100%, SH‐aLRT = 99.3%). The two individuals collected from R01 and R02 in Sakhalin, Russia, which were identified as *O. venatus* by nuclear DNA analysis using MIG‐seq (described below), belonged to the same clade as *O. similis* . There were 10 haplotypes in the *O. venatus* clade, and the COI sequences of these individuals differed by only 1–3 bp.

### 3.2 Species Identification and Genetic Diversity Based on MIG‐Seq

**FIGURE 4:**
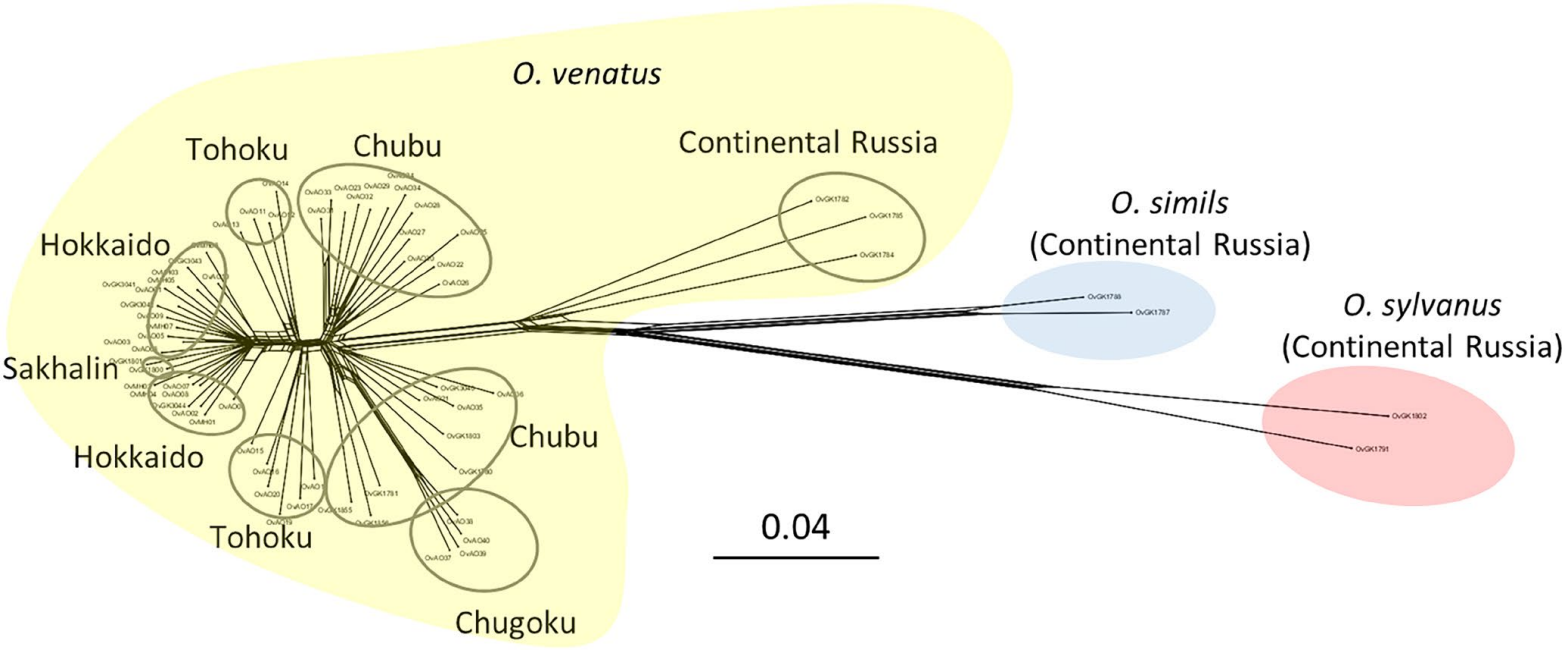
The molecular phylogenetic network tree using the NeighborNet algorithm (Bryant and Moulton 2004) concatenated 3226 SNPs data.

**FIGURE 5:**
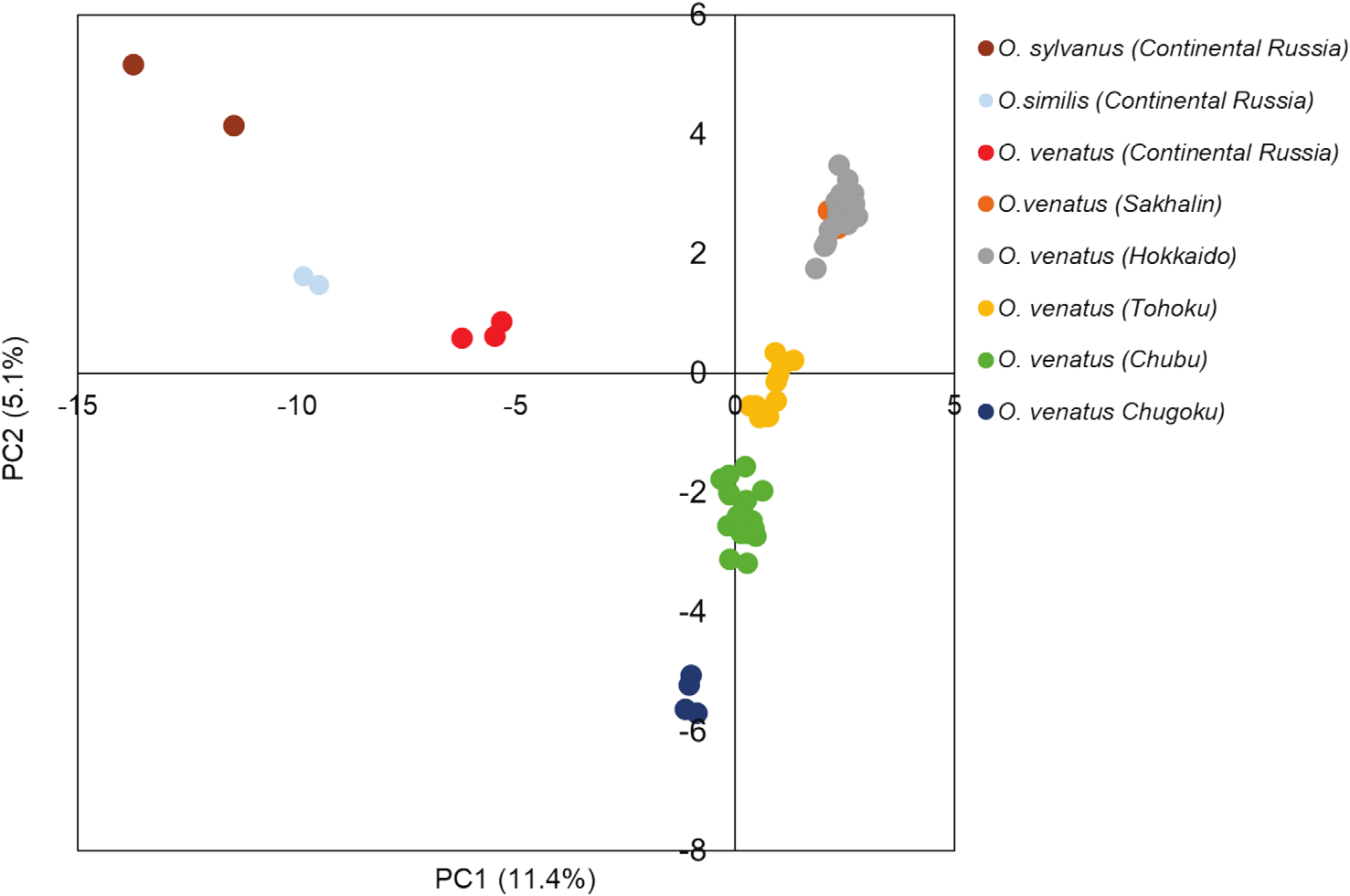
Principal component analysis plots of individuals. The data were taken from data of 3226 SNPs based on MIG‐seq analysis. Axes 1 and 2 explain 11.37% and 5.11% of the variance, respectively.

We identified 3226 SNPs in the MIG‐seq dataset from 66 individuals (missing rate: 11.14%). NeighborNet and PCA consistently showed that the two individuals from continental Russia (R03 and R05) formed a distinct group separate from all other samples (Table 1; Figures 4 and 5). They were regarded as *O. sylvanus* because they carried the *O. sylvanus* mitochondrial haplotype. Another pair of individuals from continental Russia (R08) also formed a distinct group (Table 1; Figures 4 and 5). They were regarded as *O. similis* because they carried the *O. similis* mitochondrial haplotype. Three individuals from continental Russia (two from R07 and one from R08), carrying the *O. venatus* mitochondrial haplotype, formed a cluster that was highly divergent from the *O. venatus* populations of Japan and Sakhalin. The two individuals from Sakhalin, carrying the *O. similis* mitochondrial haplotype, belonged to the same genetic cluster as the Hokkaido populations of *O. venatus*. We therefore regarded these Sakhalin individuals as *O. venatus* that had undergone introgressive hybridization with *O. similis* .

The observed and expected heterozygosity of *O. venatus* at each site in Japan were 0.040–0.055 and 0.060–0.085, respectively (Table 1). Meanwhile, the observed heterozygosity of *Ochlodes* spp. at each site in Russia ranged from 0.036 to 0.063 (Table 1). The expected heterozygosity could not be calculated as only one individual was analyzed at each site, except for R07 and R08. The expected heterozygosity for *O. venatus* at R07 and *O. similis* at R08 was 0.138 and 0.077, respectively.

### 3.3 Spatial Genetic Structure of Island Populations Based on MIG‐Seq

*O. venatus* populations in Japan and Sakhalin exhibited a significant positive correlation between geographical distance and codominant genotypic distance (*p* < 0.001, Mantel test, Figure S2). PCA and NeighborNet indicated that there were four groups in Japan, namely, Hokkaido, Tohoku, Chubu, and Chugoku groups (Figures 4 and 5).

**FIGURE 6:**
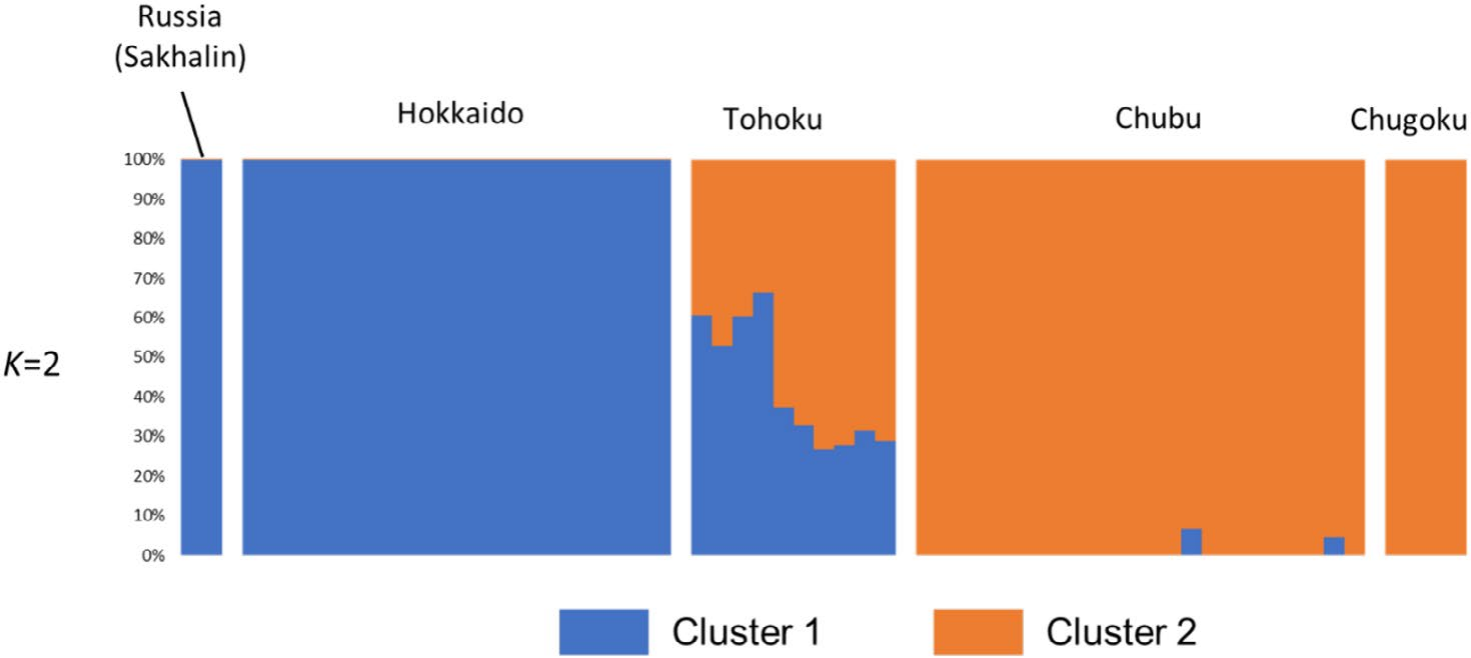
Genetic structure based on the software Admixture from 3226 SNPs (Alexander et al. 2009). The proportion of the membership coefficient of 59 individuals in Sakhalin and Japan is displayed for each of the inferred clusters (*K* = 2). Each column represents one individual.

The results of the Admixture analysis of the Japanese *O. venatus* populations at *K* = 2, which yielded the lowest CV‐error value, are illustrated in Figure 6. Cluster 1 was dominated by samples from Hokkaido and Sakhalin (the Northen Cluster), Cluster 2 was dominated by Chugoku and Chubu samples (the Western Cluster), while samples from the Tohoku district were distributed across both of these two clusters (the hybrid cluster).

### 3.4 Demography Based on MIG‐Seq

**FIGURE 7:**
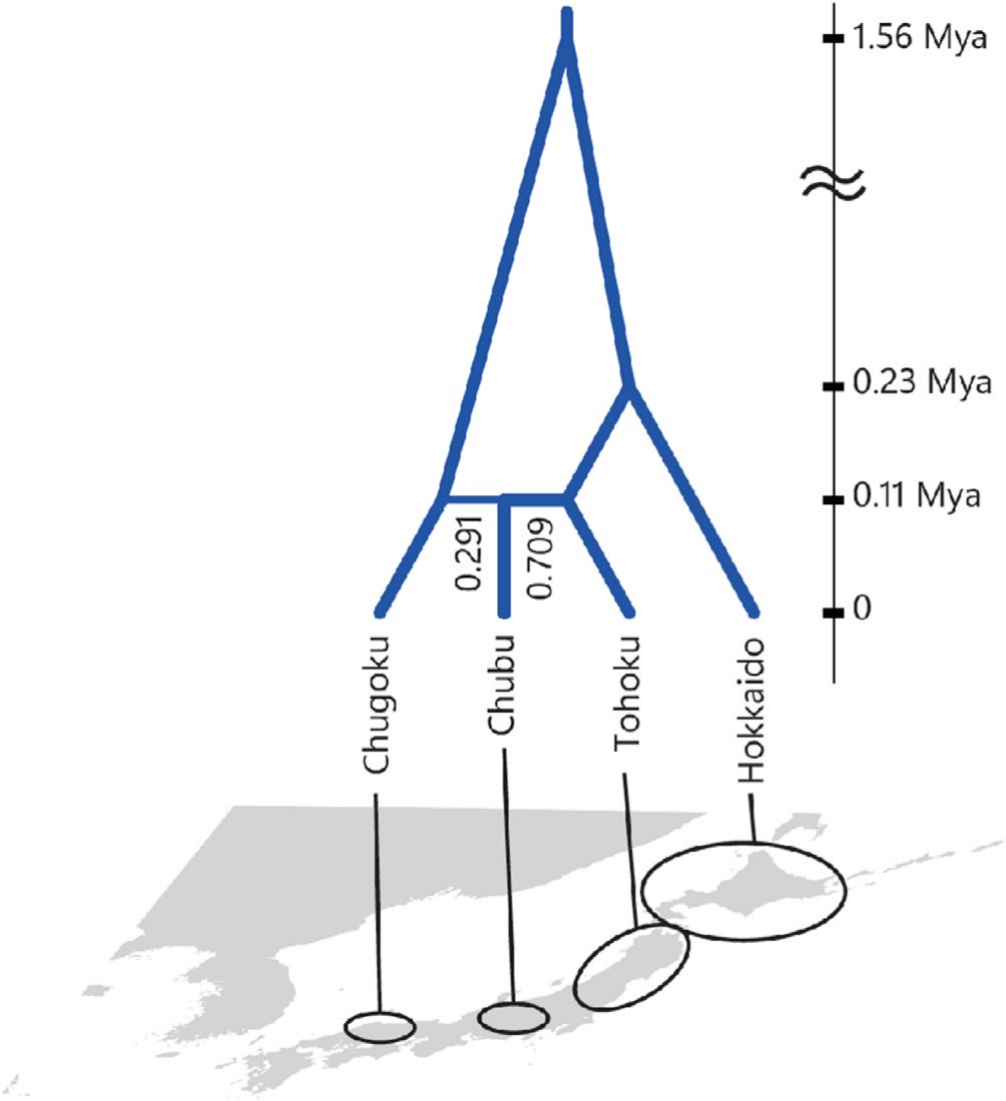
Schematic of the best demographic scenario selected using DIYABC‐RF analysis based on the data for the four populations. Competing scenarios are shown in Figure S1. Prior setting of the time of divergence and admixture events, the admixture rate during admixture events between two parental populations, and effective population size for each population are summarized in Table 2, and estimated values are given in Table 3.

We conducted an ABC analysis for the populations from Hokkaido and Honshu to draw inferences on their phylogeny and estimate their divergence times (Figure 7). By comparing 11 demographic scenarios explaining how the Hokkaido and three Honshu populations became established, we found that Scenario 10, which assumed that the Chubu population was established as an admixture between the Tohoku and Chugoku ancestral lineages, received the highest percentage of classification votes at 68.6%, resulting in a high posterior probability (PP = 0.830 with global and local errors of 0.301 and 0.170, respectively). Scenario 8 received the second‐largest number of votes (237 votes; 23.7%), whereas each of the remaining scenarios received fewer than 3% of the votes. The first divergence between the ancestral lineage of the Chugoku population and that of the other populations at t3 in Scenario 10 was estimated to have occurred 1.56 Mya (95% CI: 0.94–1.97 Mya) during the Early Pleistocene, assuming a generation time of 1 year. However, this estimate is subject to considerable uncertainty because of the limited number of samples from the Chugoku region included in the present analysis. This limitation is also reflected in the wide confidence interval for the estimated effective population size of the Chugoku population (N4 in Table 3; 95% CI: 38,679–967,309). The deep divergence between the Chugoku population and the other regional populations therefore requires further validation using larger sample sizes and more extensive genome‐wide data. Subsequently, the ancestral lineages leading to the Hokkaido and Tohoku populations diverged at t2, which was estimated to have occurred 0.23 Mya (95% CI: 0.10–0.36 Mya) during the late Middle Pleistocene. Finally, the admixture event in the Chubu population occurred at 0.11 Mya (95% CI: 0.03–0.20 Mya) around the Late Pleistocene. In this admixture event, the admixture rate from the ancestral lineage of the Tohoku population was higher than that from the Chugoku population (Figure 7, Table 3).

**TABLE 3:** Estimates of demographic parameters in Scenario 10 from demographic analysis for four populations of *O. venatus* in Japan using DIYABC‐RF.

	Expectation	Median	q0.05	q0.95
Effective population size				
N1 (Hokkaido)	664,952	634,333	342,575	1,090,620
N2 (Tohoku)	1,200,590	1,205,150	378,524	1,882,370
N3 (Chubu)	1,377,640	1,428,080	639,709	1,932,930
N4 (Chugoku)	267,937	173,910	38,679	967,309
Na	1,234,440	1,195,860	481,628	2,087,970
Nb	2,990,660	2,991,500	1,071,290	4,876,660
Nc	3,512,830	3,556,350	1,967,840	4,835,680
Time scale in generations				
t1	112,931	107,644	30,561	203,036
t2	225,029	215,987	103,820	357,387
t3	1,564,670	1,616,810	941,404	1,976,190
Admixture				
ra	0.7090	0.7308	0.5227	0.8426

Abbreviations: Nx, effective population size for each population x; q0.05 and q0.95, 5% and 95% quantiles of the posterior distribution, respectively; ra, admixture rate during an admixture event between two parental populations; tx, divergence or admixture time, with generation time as the unit.

## 4 Discussion

This study found an old, complicated history of *Ochlodes venatus* within Japan (Hokkaido and Honshu islands), and phylogenetic relationships among populations in Japan, Sakhalin and continental Russian Far East. This study also offers valuable insight into the taxonomy and distributions of its closely related species in East Asia, which are currently somewhat confused.

### 4.1 History of *O. venatus* in Japan and Russia

In Japan, the ancestral lineages of the Chugoku population and the other population (Hokkaido, Tohoku, and Chubu) diverged approximately 1.56 million years ago. The eastern Honshu (Tohoku) group then diverged from the Hokkaido group 0.23 million years ago and subsequently experienced an admixture event with the Chugoku group 0.11 million years ago (Figure 7). Because the Japanese clade is clearly distinct from other species in Eurasian Continent, we could estimate the divergence event between the ancestral lineages of Chugoku and the other populations occurred in the Japanese archipelago. It is considered that during the Ice Age, sea levels in the Tsugaru Strait were approximately 100–130 m lower than those during the interglacial period, and the width of the strait was considerably narrower (Ohshima 1991). As the Ice Age ended and transitioned into the interglacial period approximately 243,000 years ago (Lisiecki and Raymo 2005), it is suggested that the groups in Hokkaido and Tohoku diverged during this era. In addition, during the transition from the interglacial period to the glacial period, the vegetation of East Asia underwent significant changes (Bai et al. 2026). The suitable habitat for this species, whose habitat had previously been high‐altitude or high‐latitude grasslands during the interglacial period, shifted to the lowlands of western Japan, and it is possible that populations from Chubu and Chugoku groups interbred. Scenario 10 was reasonably well supported, with a posterior probability of 0.830 and substantially more classification votes than the alternative scenarios. Nevertheless, the global prior error rate of 0.301 and local posterior error of 0.170 suggest that some uncertainty remains in distinguishing among the competing demographic histories, particularly given the uneven sample sizes among populations. In addition, it is noted that our findings cannot rule out the occurrence of more than one admixture event between the Chugoku and the other Honshu (Tohoku and Chubu) groups since the DIYABC assumes a simple prior model.

Notably, the occurrence of divergence and admixture within Japan suggests that *O. venatus* was distributed in the Hokkaido, Tohoku, and Chubu regions of Japan at least 200,000 years ago, and at least 100,000 years ago in the Chugoku region. Several studies have examined the time of divergence of grassland species between the Eurasian continent and the Japanese archipelago, yielding ancient dates. In butterflies, for example, it is estimated that *Aporia hippia* diverged from the continent approximately 80,000 years ago, while *Melitaea ambigua* did so approximately 72,000 years ago (Nakahama et al. 2018; Nakatani et al. 2020). In herbal plants, *Geranium soboliferum var. kiusianum* is estimated to have diverged from the continent 9390–47,300 years ago (Kurata et al. 2019), *Inula britannica* approximately 25,200 years ago (Sakaba et al. 2023), and *Viola orientalis* 29,400–58,800 years ago (Sata et al. 2021). Considering the assertion that humans first colonized the Japanese archipelago approximately 32,000–38,000 years ago, it is likely that grassland butterflies, including *O. venatus*, were distributed in Japan long before humans first arrived there. In particular, the date of colonization by *O. venatus* is longer ago than the previously reported dates for *A. hippia* and *M. ambigua* , suggesting that grassland butterflies could have persisted during the previous interglacial period with a warm and humid climate, which is detrimental to the maintenance of grasslands, and have a longer history in Japan than previously thought.

However, from the mitochondrial DNA results, *O. venatus* did not form distinct clades that differed between Eurasia (including Europe, Russia, and China) and the Japanese archipelago, and base substitutions were only 1–3 bp in length. Thus, we could not estimate divergence times among these regions. We do not know why the mitochondrial DNA results did not reveal the existence of distinct clades in the present study. The difference in substitution rate and maternal inheritance of mitochondrial DNA may have yielded different results compared with the equivalent findings for nuclear genome‐wide SNPs.

### 4.2 Phylogeny and Distribution of Venatus‐Complex in Far‐East Asia

The individuals from continental Russia in this study were divided into three groups based on MIG‐seq (Figures 4 and 5), which corresponded to *O. sylvanus* (sites R03 and R05), *O. similis* (R08), or *O. venatus* (R07 and R08) based on the sequences of the mitochondrial DNA COI region (Figure 3, Table 1). This concordance indicates that molecular species identification based on the mitochondrial DNA COI region is consistent with that obtained from genome‐wide SNPs by MIG‐seq for the Russian samples examined in this study, although our sampling coverage was limited to part of the Russian Far East and no samples from China and Korea were available for comparison. In contrast, the nuclear SNPs of the two individuals from Sakhalin coincided with those from the Hokkaido population of *O. venatus* (Figures 4 and 5), while the mitochondrial COI regions of these two individuals were classified as the *O. similis* clade (Figure 3). This suggests that the Sakhalin *O. venatus* population would have experienced introgressive hybridization with *O. similis* in the past, replacing the *O. venatus* mitochondrial haplotype with that of *O. similis* . However, because of small sample size from Sakhalin, further analysis is necessary with a larger sample size in order to determine the molecular phylogenetic position of the Sakhalin population and the potential for interspecific hybridization.

It should be noted that the taxonomy and distributions of *O. venatus* and its related species (*venatus‐*complex) have a long history of revision. Although *O. venatus* was once thought to be distributed from Europe to East Asia, including Japan (Farahpour‐Haghani et al. 2023), it is now divided into two species; *O. sylvanus* in the west and *O. venatus* in eastern Eurasia, bounded by the Taklamakan and Gobi deserts (Chiba and Tsukiyama 1996; Shirôzu 2006). Chiba and Tsukiyama (1996) proposed the following four species as *venatus‐*complex; *O. venatus*, *O. similis* , *O. saggita*, and *O. hyrcana* (now *O. sylvanus*), based on differences in wing spots and genitalia. Recent studies based on the mitochondrial COI barcode region have revealed that these species are genetically distinct from each other (Farahpour‐Haghani et al. 2023; Zhu et al. 2023). However, likely in part due to the taxonomic confusion, the distributions of these related species remain unresolved in continental East Asia. While our genetic analysis detected *O. sylvanus* and *O. similis* in the continental Russian Far East, only *O. venatus* is recorded in the Russian literature (Gorbunov and Kosterin 2003). The finding of *O. sylvanus* in the eastern part of the Eurasian continent is unexpected since its distribution is considered to be in western Eurasia (Chiba and Tsukiyama 1996). In Korea, while one pictorial book illustrated *O. venatus* and *O. similis* (Kim and So 2012), another illustrated *O. venatus* and *O. sylvanus* (Paek and Shin 2014). Given the possibility of introgression between *O. venatus* and *O. similis* , as observed in the Sakhalin individuals in this study, future molecular phylogenetic analyses of the *venatus‐*complex in East Asia using both nuclear and mitochondrial DNA are necessary to clarify the distributions, taxonomy, and evolutionary relationships of these three species.

### 4.3 Implications for Conservation

This study supports the historical antiquity and conservation value of Japanese grassland butterflies, as previously suggested by Ohwaki (2018). *Ochlodes venatus* diverged into the Japanese archipelago even longer ago than suggested in previous studies (Nakahama et al. 2018; Nakatani et al. 2020). It is also likely that the origin of the populations differs between northern and western Japan, which may reflect the species' long and complex history. Although it has been considered that this species and other grassland butterflies have been heavily influenced by humans, their history predates human colonization in Japan. Meanwhile, it is widely believed that, without human activity, grasslands would have been almost completely absent during the warm and humid interglacial periods before the last glacial period (Numata 1969; Fukushima 2017). However, this is contradicted by the persistence of this grassland species in the Japanese archipelago during the last glacial period and the previous interglacial period. Even in the absence of human activity, various natural disturbances may have contributed to the persistence of grassland butterflies during the warm interglacial period. This would highlight the importance of grassland butterflies as well as forest butterflies as valuable components of the Japanese biota and their status as potentially being of great conservation value.

Based on the results of nuclear genome‐wide SNPs obtained by MIG‐seq, *O. venatus* was divided into four groups in Japan: those in the Hokkaido, Tohoku, Chubu, and Chugoku regions. At present, this species is not listed as endangered in Japan, and no conservation action is currently being undertaken to protect it. However, if further declines occur and conservation measures become necessary, it is suggested that the four groups from Hokkaido, Tohoku, Chubu, and Chugoku should be considered as independent conservation units. It should be noted that there is a high risk of genetic disturbance if individuals are translocated across these conservation units. Particularly in the Chugoku region, this species is on the Red List in all prefectures except Hiroshima Prefecture, with many populations facing extinction (search system of Japanese Red Data, http://jpnrdb.com/index.html). Consequently, conservation priorities in Chugoku may be higher than elsewhere. This research should thus provide useful insights for measures to conserve this species.

It is noted that, in insects with short generation times such as butterflies, rapid population declines can lead to significant changes in allele frequency due to genetic drift (Nakahama and Isagi 2018). Therefore, estimating genetic diversity and spatial genetic structure simultaneously, when the risk of extinction is low, as in this species, would be extremely helpful for constructing future conservation strategies.

## Author Contributions

**Naoyuki Nakahama:** conceptualization (lead), data curation (lead), formal analysis (equal), funding acquisition (equal), investigation (lead), methodology (lead), project administration (equal), resources (equal), software (lead), supervision (equal), validation (equal), visualization (lead), writing – original draft (lead), writing – review and editing (lead). **Gohta Kinoshita:** formal analysis (equal), methodology (equal), writing – review and editing (equal). **Masato Hayamizu:** resources (equal), validation (equal), writing – review and editing (equal). **Kazusa Nishimura:** methodology (equal), validation (equal), writing – review and editing (equal). **Seiya Kudo:** resources (equal), validation (equal), writing – review and editing (equal). **Yuriy Tshistjakov:** resources (equal), validation (equal), writing – review and editing (equal). **Atsushi Ohwaki:** conceptualization (supporting), funding acquisition (lead), resources (equal), supervision (equal), validation (equal), writing – review and editing (equal).

## Funding

This study was financially supported by Grant‐in‐Aid for Scientific Research of Japan Society for the Promotion of Science (21H02221/23K21209).

## Conflicts of Interest

The authors declare no conflicts of interest.

## Supporting information


**Figure S1:** Graphic representation of alternative demographic models for *Ochlodes venatus* in Japan. Four population groups were defined based on each district. Different colors in population trees indicate different population sizes.
**Figure S2:** The relationships between Codominant genotypic distance based on 3226 SNPs data and geographic distances at the individual level.

## Data Availability

Our sequences were registered in GenBank (accession numbers: PZ264059–PZ264126). MIG‐seq data are available at PRJNA1450204.
