# Supplementary material for: Phylogeography of the Grassland Skipper Ochlodes venatus Supports the Hypothesis That This Japanese Grassland Insect Has a Long History
Source: Ecol Evol. 2026 Aug 18;16(8):e74193. doi: 10.1002/ece3.74193 (PMC13482905; doi:10.1002/ece3.74193)
Supplement: Supplementary file 1 [file ECE3-16-e74193-s001.docx]

**Fig. S1.**　Graphic representation of alternative demographic models for *Ochlodes venatus* in Japan. Four population groups were defined based on each district. Different colours in population trees indicate different population sizes.

**Fig. S2.** The relationships between Codominant genotypic distance based on 3226 SNPs data and geographic distances at the individual level.
